# Supplementary material for: N-terminal half of MED14 is critical for Mediator-RNA polymerase II interaction and the resulting transcription
Source: J Biol Chem. 2025 Oct 17;301(12):110837. doi: 10.1016/j.jbc.2025.110837 (PMC12719649; doi:10.1016/j.jbc.2025.110837)
Supplement: Supplementary_Figures 1-5 [file mmc2.docx]

Supplementary Table S2

Protein ID: Q_9079277 *S. pombe* MED14

| AA_QUERY | 1 | **MEPPAIPHITEGFYPLPEIVETFSHHVLQELVSLAEVLPSMSNVEKKKKILDWLLRSRAFTMRLLVLARWVHLSPSVHRCIDVVAFLQGQ**    90 |
| --- | --- | --- |
| SS_PSIPRED |  | HHHHHHHHHHHHHHHHHHHHHHH    HHHHHHHHHHHHHHHHHHHHHHHHHHHHHH HHHHHHHHHHHHHHHHH |
| SS_SPIDER2 |  | EEHHHHHHHHHHHHHHHHHHHHHH      HHHHHHHHHHHHHHHHHHHHHHHHHHHHH  HHHHHHHHHHHHHHHH |
| SS_PSSPRED |  | HHHHHHHHHHHHHHHHHHHHHHHHH  HHHHHHHHHHHHHHHHHHHHHHHHHHHHH   HHHHHHHHHHHHH |
| SS_DEEPCNF |  | HHHHHHHHHHHHHHHHHHHHH       HHHHHHHHHHHHHHHHHHHHHHHHHHHH   HHHHHHHHHHHHH |
| CC_COILS_W28 |  |  |
| CC_PCOILS_W28 |  | CCCC |
| DO_DISOPRED3 |  | DDD |
| DO_SPOTD |  | DD |
| DO_IUPRED |  |  |
|  | | |
|  | | |
|  | | |
|  | | |
|  | | |
| AA_QUERY | 91 | **KFCFQNLVHVLQDIRYQLSFARLRNSDLVTALDILSTGTSLRLANAPTSKLYMLSESPLSTKQILQTLHALNMLIRIRLSLYEIIPTPFQ**    180 |
| SS_PSIPRED |  | HHHHHHHHHHHHHHHHH HHH      HHHHHHHH                         HHHHHHHHHHHHHHHHHHH       HHHH |
| SS_SPIDER2 |  | HHHHHHHHHHHHHHHHHHHH          HHHHHH   E                    HHHHHHHHHHHHHHHHHHH       H HE |
| SS_PSSPRED |  | HHHHHHHHHHHHHHHHHHHHHHHH  HHHHHHHHHH   EEEE      EEEEE      HHHHHHHHHHHHHHHHHHHHHHHH |
| SS_DEEPCNF |  | HHHHHHHHHHHHHH            EEEEEE               EEEE      HHHHHHHHHHHHHHHHHHHHH        E |
| CC_COILS_W28 |  | CCCCCCCCCCCCCCCCCCCCCCCCCCCCC |
| CC_PCOILS_W28 |  | CCCCCCCCCCCCCCCCCCCCCCCCCCCCCCCCCCCCCC |
| DO_DISOPRED3 |  |  |
| DO_SPOTD |  |  |
| DO_IUPRED |  |  |
|  | | |
|  | | |
|  | | |
|  | | |
|  | | |
| AA_QUERY | 181 | **HFTIANGRCTFTVPNEFSVSLTTNSQDPKSTGISFQWIVVDFQFHLPDFSSTPAKYRVFIELHLNEEIAAAFVLQKPILPLIYNILHKFC**    270 |
| SS_PSIPRED |  | EEE   EEEE    EEEEEEE             EEEEEEEEE        HHHHHHHHHHHHHHHHHHHHH   HHHHHHHHHHHHH |
| SS_SPIDER2 |  | EEEEE  EEEEEE  EEEEEEEE            EEEEEEEEEE        HHHHHHHHHHHHHHHHHHHH     HHHHHHHHHHHH |
| SS_PSSPRED |  | EEEE  EEEEEE  EEEEEEE           EEEEEEEEEEEE         EEEEEEEEE  HHHHHHHHHH HHHHHHHHHHHHHH |
| SS_DEEPCNF |  | EEEEE  EEEEEE  EEEEEEEE         EEEEEEEEEEEEE         EEEEEEEEEE HHHHHHHH     HHHHHHHHHHHH |
| CC_COILS_W28 |  |  |
| CC_PCOILS_W28 |  |  |
| DO_DISOPRED3 |  |  |
| DO_SPOTD |  |  |
| DO_IUPRED |  |  |
|  | | |
|  | | |
|  | | |
|  | | |
|  | | |
| AA_QUERY | 271 | **LYQRLNLLSQQTFQLSRESWLGHLRGVYDEKPPRLRLYYWPQLNVKKEGKPGKIGHYIHIFVNTQPISAFERTLSSKRSSCEYDHFLLLV**    360 |
| SS_PSIPRED |  | HHHHHHHHHHHHHHHHH     EEEEEE     EEEEEE                 EEEEEEE     HHHH              EEEE |
| SS_SPIDER2 |  | HHHHHHHHHHHHHHHHH      EEEEE     EEEEEE                 EEEEEEE     HHHHH             EEEE |
| SS_PSSPRED |  | HHHHHHHHHHHHHHHHHHHHHHHHH         EEEEE                EEEEEEE     HHHHHHHH         EEEEEE |
| SS_DEEPCNF |  | HHHHHHHHHHHHHHHHH                EEEEEEE                EEEEEEE     HHHHHH          EEEEEE |
| CC_COILS_W28 |  |  |
| CC_PCOILS_W28 |  |  |
| DO_DISOPRED3 |  |  |
| DO_SPOTD |  | DDDDDDDD                     DDDDDDD |
| DO_IUPRED |  |  |
|  | | |
|  | | |
|  | | |
|  | | |
|  | | |
| AA_QUERY | 361 | **EWHHDGIVEHVPLDDHMDAQHLLLLITQKHAQLILEQIRKELHPNIFSEHVGGGLKIHVFDNEIIVKVNSVTGRLVLSSSASPLSPPRHL**    450 |
| SS_PSIPRED |  | EEEE   EE         HHHHHHHHHHHHHHHHHHHHHHHH           EEEEEE   EEEEEEE    EEEEE          HH |
| SS_SPIDER2 |  | EE     EE         HHHHHHHHHHHHHHHHHHHHHHHH    EE      EEEEEE  EEEEEEE    EEEEEE        HHH |
| SS_PSSPRED |  | EEE    EEE        HHHHHHHHHHHHHHHHHHHHHHHH   HHHHH    EEEEEE  EEEEEEEEEE EEEEE        HHHH |
| SS_DEEPCNF |  | EEE   EEEEEE      HHHHHHHHHHHHHHHHHHHHHHHH            EEEEE   EEEEEE     EEEE          HHH |
| CC_COILS_W28 |  |  |
| CC_PCOILS_W28 |  |  |
| DO_DISOPRED3 |  |  |
| DO_SPOTD |  |  |
| DO_IUPRED |  |  |
|  | | |
|  | | |
|  | | |
|  | | |
|  | | |
| AA_QUERY | 451 | **RAAEKNIALNTQPPAQILNRLYFFCIQTQLLEVAQCAELHAVQGYYSFPYLTFSKGKWRKDGDSLWVLAYNVESNSWSVRLLNAAGQTLY**    540 |
| SS_PSIPRED |  | HHHHHHH      HHHHHHHHHHHHHHHHHHHHHHH   EE        EEEE          EEEEEEEE    EEEEEEE     EEE |
| SS_SPIDER2 |  | HHHHHHHH     HHHHHHHHHHHHHHHHHHHHHHH   EEEE      EE     H       EEEEEEEE   EEEEEEE     EEE |
| SS_PSSPRED |  | HHHHHHHE     HHHHHHHHHHHHHHHHHHHHHHHHHHHHHH       EEE          EEEEEEEEE    EEEHHHHHH  EEE |
| SS_DEEPCNF |  | HHH          HHHHHHHHHHHHHHHHHHHHH               EEEEEEEEEEE   EEEEEEEEE   EEEEEEEE    EEE |
| CC_COILS_W28 |  |  |
| CC_PCOILS_W28 |  |  |
| DO_DISOPRED3 |  |  |
| DO_SPOTD |  |  |
| DO_IUPRED |  | D D |
|  | | |
|  | | |
|  | | |
|  | | |
|  | | |
| AA_QUERY | 541 | **TQDVHTTKGTLSIESFSRLSYLLEVQILLFNVQTACQARGMPFEYLPIPPKALIEDDFTTYVQTGCLCIMMPSSNEDMLPVVFVRAHDGQ**    630 |
| SS_PSIPRED |  | EEEE        HHHHHHHHHHHHHHHHHHHHHHHHHH                   EEEEE     HH         EEEEEEE    E |
| SS_SPIDER2 |  | EEE         HHHHHHHHHHHHHHHHHHHHHHHHHH                             E E         EEEEEE    E |
| SS_PSSPRED |  | EEEEHHHHHHHHHHHHHHHHHHHHHHHHHH           HHH    HHHHHHH EEEEE         EEEEEEE |
| SS_DEEPCNF |  | EEE         HHHHHHHHHHHHHHHHHHHHHHHHHH                    HHHHHH  EEEE       EEEEEEEEE   E |
| CC_COILS_W28 |  |  |
| CC_PCOILS_W28 |  |  |
| DO_DISOPRED3 |  |  |
| DO_SPOTD |  |  |
| DO_IUPRED |  |  |
|  | | |
|  | | |
|  | | |
|  | | |
|  | | |
| AA_QUERY | 631 | **LIFDSRIKGKLPYQSETETEKNCYIDWRTGRITIRVQNFSSFEKTWIGLLKLVALSKTSAFNVDCITLKHVDFTYLDDEKFRATIHDDNT**    720 |
| SS_PSIPRED |  | EEEEEEEEE             EEEE    EEEEEE  HHHHHHHHHHHHHHHHHHHH   EEEEEE  EEEEEE     EEEEEE |
| SS_SPIDER2 |  | EEEEEEEE E             EEE    EEEEEE  HHHHHHHHHHHHHHHHHHHH   E EEE   EEEEEE     EEEEEE |
| SS_PSSPRED |  | EEEE                  EEEEEE  EEEEEEE    HHHHHHHHHHHHHHH     EEEEEEE          HHHEEEE |
| SS_DEEPCNF |  | EEEEEEEEEE            EEEE    EEEEEE     HHHHHHHHHHHHEE       EEEEEEEEEEEE       EEEEEE  E |
| CC_COILS_W28 |  |  |
| CC_PCOILS_W28 |  |  |
| DO_DISOPRED3 |  |  |
| DO_SPOTD |  |  |
| DO_IUPRED |  |  |
|  | | |
|  | | |
|  | | |
|  | | |
|  | | |
| AA_QUERY | 721 | **FTLHFFNRHSPFHLISQFLQDTFSDGPSAIQPLRVIMDRTRGVLVAQELGYVVLARSLRQYRIILSKNHGIQVLLNRHGCILQDLSYLSA**    810 |
| SS_PSIPRED |  | EEEE      HHHHHHHHHHHH   HHHHHHHHHHHHHHHHHHHH    EEEEEE   EEEEEE   EEEEEEEEE   EEEEEEEE |
| SS_SPIDER2 |  | EEEEE       HHHHHHHHHHHH  HHHHHHHHHHHHHHHHHHHH   EEEEEEE   EEEEEE    EEEEEEE    EEEEEEEEE |
| SS_PSSPRED |  | EEEEEE     HHHHHHHHHHHH     HH HHHHHHHH   EEEHHHH HHHHHHHHHHHHHHHH     EEEEHHH HHHHHHHHH |
| SS_DEEPCNF |  | EEEEEE    HHHHHHHHHHHHH        HHHHHHHH           EEEEE     EEEEEE   HHHHHH     EEEEEEEE |
| CC_COILS_W28 |  |  |
| CC_PCOILS_W28 |  |  |
| DO_DISOPRED3 |  |  |
| DO_SPOTD |  |  |
| DO_IUPRED |  |  |
|  | | |
|  | | |
|  | | |
|  | | |
|  | | |
| AA_QUERY | 811 | **DSRYLEGTQTLTSQWEPCSWLNTVWEGDLGDDELNGQIEAAPEMHLIKMNKTADLTAILKRILAISRKK**    879 |
| SS_PSIPRED |  | HHH   EE           EEE     EEE   HH HHHHHHHHHHH |
| SS_SPIDER2 |  | HHHHHHHH            E    HHHHHHH     HHHHHHHHH |
| SS_PSSPRED |  | HHHHHH                 HHHHEE    HHHHHHHHHHHHHH |
| SS_DEEPCNF |  | EE                     EE     EEE      HHHHHHHHHHH |
| CC_COILS_W28 |  |  |
| CC_PCOILS_W28 |  |  |
| DO_DISOPRED3 |  | DDDD  DDD |
| DO_SPOTD |  | DD |
| DO_IUPRED |  |  |
|  | | |
|  | | |
|  | | |
|  | | |
|  | | |

SS = Secondary Structure; H = Alpha-helix; E= Beta-strand; CC = Coiled coils; D, DO = Disordered Region

Protein ID: Q_7346414 MED14 Human

| AA_QUERY | 1 | **MAPVQLENHQLVPPGGGGGGSGGPPSAPAPPPPGAAVAAAAAAAASPGYRLSTLIEFLLHRAYSELMVLTDLLPRKSDVERKIEIVQFAS**    90 |
| --- | --- | --- |
| SS_PSIPRED |  | HHHHHHHHHHH   EEHHHHHHHHHHHHHHHHHHHHHH     HHHHHHHHHHHHH |
| SS_SPIDER2 |  | HHHHHHH     EEHHHHHHHHHHHHHHHHHHHHHH      HHHHHHHHHHHH |
| SS_PSSPRED |  | EEE                           HHHHHHHHHHH     HHHHHHHHHHHHHHHHHHHHHHH      HHHHHHHHHHHH |
| SS_DEEPCNF |  | HHHHHHHHH       HHHHHHHHHHHHHHHHHHHHH       HHHHHHHHHHHH |
| DO_DISOPRED3 |  | DDDD DDDDDDDDDDDDDDDDDDDDDDDDDDDDDDDDDDDDD |
| DO_SPOTD |  | DDDDDDDDDDDDDDDDDDDDDDDDDDDDDDDDDDDDDDDDDDDD |
| DO_IUPRED |  | DDDDDDDDDDDDDDDDDDDDDDDDDDDDDDDDDD |
|  | | |
|  | | |
|  | | |
|  | | |
|  | | |
| AA_QUERY | 91 | **RTRQLFVRLLALVKWANNAGKVEKCAMISSFLDQQAILFVDTADRLASLARDALVHARLPSFAIPYAIDVLTTGSYPRLPTCIRDKIIPP**    180 |
| SS_PSIPRED |  | HHHHHHHHHHHHHHHHH  HHHHHHHHHHHHHHHHHHHHHHHHHHHHHHHHHHHHH           EEE            HH |
| SS_SPIDER2 |  | HHHHHHHHHHHHHHHH   HHHHHHHHHHHHHHHHHHHHHHHHHHHHHHHHHHHHH       HHHHHHHHH        HHHH |
| SS_PSSPRED |  | HHHHHHHHHHHHHHHHHH   HHHHHHHHHHH HHHHHHHHHHHHHHHHHHHHHHHH    HHHHHHEEEEE         HHH |
| SS_DEEPCNF |  | HHHHHHHHHHHHHHHHH     HHHHHHHHH     EEEEE HHHHHHHHHHHHHHH        EEEEEE          HHH |
| DO_DISOPRED3 |  |  |
| DO_SPOTD |  |  |
| DO_IUPRED |  |  |
|  | | |
|  | | |
|  | | |
|  | | |
|  | | |
| AA_QUERY | 181 | **DPITKIEKQATLHQLNQILRHRLVTTDLPPQLANLTVANGRVKFRVEGEFEATLTVMGDDPDVPWRLLKLEILVEDKETGDGRALVHSMQ**    270 |
| SS_PSIPRED |  | HHHHHHHHHHHHHHHHHHHHH    HHHH  EEE  EEEEEE  EEEEEEEE        EEEEEEEEEEEE           HHH |
| SS_SPIDER2 |  | HHHHHHHHHHHHHHHHHHH      HHHEEEEEE  EEEEEE  EEEEEEEEE       EEEEEEEEEEE            HHH |
| SS_PSSPRED |  | HHHHHHHHHHHHHHHHHHHH     HHH  EEE   EEEEEEE EEEEEEEEE      HHHHHHHHHEE        HHEHHHHH |
| SS_DEEPCNF |  | HHHH   HHHHHHHHHHHH            EEE  EEEEEEEEEEEEEEEEE         EEEEEEEEE        HHHHHHH |
| DO_DISOPRED3 |  |  |
| DO_SPOTD |  |  |
| DO_IUPRED |  |  |
|  | | |
|  | | |
|  | | |
|  | | |
|  | | |
| AA_QUERY | 271 | **ISFIHQLVQSRLFADEKPLQDMYNCLHSFCLSLQLEVLHSQTLMLIRERWGDLVQVERYHAGKCLSLSVWNQQVLGRKTGTASVHKVTIK**    360 |
| SS_PSIPRED |  | HHHHHHHHHHHH     HHHHHHHHHHHHHHHHHHHHHHHHHHHHHHHHHHH EEEEEE    EEEEEEEE            EEEEEEE |
| SS_SPIDER2 |  | HHHHHHHHHH      HHHHHHHHHHHH  HHHHHHHHHHHHHHHHH      EEEEEE    EEEEEEEE            EEEEEEE |
| SS_PSSPRED |  | HHHHHHHHHHHHH    HHHHHHHHHHHHHHHHHHHHHHHHHHHHHHHHH   EEEEHHH   EEEEEEE HHH       EEEEEEEEE |
| SS_DEEPCNF |  | HHHHHHHHHH        HHHHHHHHHHHHHHHHHHHHHHHHHHHHHHHH   EEEEEEE   EEEEEEEE           EEEEEEEE |
| DO_DISOPRED3 |  |  |
| DO_SPOTD |  |  |
| DO_IUPRED |  |  |
|  | | |
|  | | |
|  | | |
|  | | |
|  | | |
| AA_QUERY | 361 | **IDENDVSKPLQIFHDPPLPASDSKLVERAMKIDHLSIEKLLIDSVHARAHQKLQELKAILRGFNANENSSIETALPALVVPILEPCGNSE**    450 |
| SS_PSIPRED |  | E        EEEEE         HHHHHH       HHHHHHHHHHHHHHHHHHHHHHHHHH       EE     EEEEEE |
| SS_SPIDER2 |  | E         EEEE     H   HHHHHH       HHHHHHHHHHHHHHHHHHHHHHHHH                EEEE E      E |
| SS_PSSPRED |  | E        HHH          HHHHHHHHHH  HHHHHHHHHHHHHHHHHHHHHHHHHHHH       HHHHHHHHHHHHH       H |
| SS_DEEPCNF |  | E        EEEE         HHHHHHHH      HHHHHHHHHHHHHHHHHHHHHHHHH                EEEEEE |
| DO_DISOPRED3 |  |  |
| DO_SPOTD |  |  |
| DO_IUPRED |  |  |
|  | | |
|  | | |
|  | | |
|  | | |
|  | | |
| AA_QUERY | 451 | **CLHIFVDLHSGMFQLMLYGLDQATLDDMEKSVNDDMKRIIPWIQQLKFWLGQQRCKQSIKHLPTISSETLQLSNYSTHPIGNLSKNKLFI**    540 |
| SS_PSIPRED |  | EEEEEEE    EEEEEE    HHHHHHHHHHHH  HHHHHHHHHHHHHHHHHHHHHHHHHH   EEE                   EEEE |
| SS_SPIDER2 |  | EEEEEE     EEEEEE    HHHHHHHHHHH   HHHHHHHHHHHHHHHHHHHHHHHHH    EEEEE                 EEEE |
| SS_PSSPRED |  | HHHHHHHHHHHHHHHHHH     HHHHHHHHHHHHHHHHHHHHHHHHHHHHHHHHHHHHHH        EE               HHHH |
| SS_DEEPCNF |  | EEEEEEE    EEEEEEE     HHHHHHHHHHHHHHHHHHHHHHHHHHHHHHHHHHHH           E               EEEE |
| DO_DISOPRED3 |  |  |
| DO_SPOTD |  |  |
| DO_IUPRED |  |  |
|  | | |
|  | | |
|  | | |
|  | | |
|  | | |
| AA_QUERY | 541 | **KLTRLPQYYIVVEMLEVPNKPTQLSYKYYFMSVNAADREDSPAMALLLQQFKENIQDLVFRTKTGKQTRTNAKRKLSDDPCPVESKKTKR**    630 |
| SS_PSIPRED |  | EE     EEEEEEEEE     EEEEEEEEEEEEE       HHHHHHHHHHHHHH   EEE |
| SS_SPIDER2 |  | EE     EEEEEEE        EEEEEEEEEEEEE      HHHHHHHHHHHHHHHH  EE                            H |
| SS_PSSPRED |  | HHHH   EEEEEEEEE       EEEEEEEEEEE       HHHHHHHHHHHHHHHHHHHH         HHHH              HH |
| SS_DEEPCNF |  | EE     EEEEEEEEE      EEEEEEEEEEEE       HHHHHHHHHHHHHH    E |
| DO_DISOPRED3 |  | DDDDDDDDDDDDDDDDDDDDDDDD |
| DO_SPOTD |  | DDDDDDDDDDDDDDDDDDDDDDDDD |
| DO_IUPRED |  | DDD    DDDDDDDDDDDDDDD |
|  | | |
|  | | |
|  | | |
|  | | |
|  | | |
| AA_QUERY | 631 | **AGEMCAFNKVLAHFVAMCDTNMPFVGLRLELSNLEIPHQGVQVEGDGFSHAIRLLKIPPCKGITEETQKALDRSLLDCTFRLQGRNNRTW**    720 |
| SS_PSIPRED |  | HHHHHHHHHHHHHH     HHHHHHHHHH        EE    EEEEEEE          HHHHHHHHHHH EEEEEE     EEE |
| SS_SPIDER2 |  | HHHHHHHHHHHHHHHH     HHH HHH       E EEE     EEEEEE          HHHHHHHHHH EEEEEEE      EE |
| SS_PSSPRED |  | HHHHHHHHHHHHHHHHHH        EEEEE         EEEE   HHHHHHHH         HHHHHHHHHHHHHHEEEE     HHH |
| SS_DEEPCNF |  | HHHHHHHHHHH     EEEEEEEE         EEE     EEEEEEEE        HHHHHHHHH     EEEEE     EE |
| DO_DISOPRED3 |  | DD |
| DO_SPOTD |  | D |
| DO_IUPRED |  |  |
|  | | |
|  | | |
|  | | |
|  | | |
|  | | |
| AA_QUERY | 721 | **VAELVFANCPLNGTSTREQGPSRHVYLTYENLLSEPVGGRKVVEMFLNDWNSIARLYECVLEFARSLPDIPAHLNIFSEVRVYNYRKLIL**    810 |
| SS_PSIPRED |  | EEEEEEE              EEEEEEEEE          HHHHHHHHHHHHHHHHHHHHHHHHHH           EEEEEEE  EEEE |
| SS_SPIDER2 |  | EEEEEEE               EEEEEEEEEE         HHHHHHHHHHHHHHHHHHHHHHHHH       H  EEEEEEEE  EEEE |
| SS_PSSPRED |  | HHHHHHH                EEEEEHHHHH      HHHHHHHHHHHHHHHHHHHHHHHHHHH     HHHHHH EEEHHHHHHHHH |
| SS_DEEPCNF |  | EEEEEEE                EEEEEE           EEEEEE   HHHHHHHHHHHHHHHHH            EEEE    EEEE |
| DO_DISOPRED3 |  |  |
| DO_SPOTD |  |  |
| DO_IUPRED |  |  |
|  | | |
|  | | |
|  | | |
|  | | |
|  | | |
| AA_QUERY | 811 | **CYGTTKGSSISIQWNSIHQKFHISLGTVGPNSGCSNCHNTILHQLQEMFNKTPNVVQLLQVLFDTQAPLNAINKLPTVPMLGLTQRTNTA**    900 |
| SS_PSIPRED |  | EEE     EEEEEEE    EEEEEE            HHHHHHHHHHHH     HHHHHHHHHH HHHHHHHH |
| SS_SPIDER2 |  | EEE    EEEEEEEE    EEEEEEEE          HHHHHHHHHHHH     HHHHHHHHHH HHHHHHH |
| SS_PSSPRED |  | HH      EEEEEE HH HEEEEEE           HHHHHHHHHHHHHH   HHHHHHHHHHH    HHHHH       E       HH |
| SS_DEEPCNF |  | EEE     EEEEEE     EEEEEE           HHHHHHHHHHHHHH    HHHHHHHHHH    HHHH |
| DO_DISOPRED3 |  |  |
| DO_SPOTD |  |  |
| DO_IUPRED |  |  |
|  | | |
|  | | |
|  | | |
|  | | |
|  | | |
| AA_QUERY | 901 | **YQCFSILPQSSTHIRLAFRNMYCIDIYCRSRGVVAIRDGAYSLFDNSKLVEGFYPAPGLKTFLNMFVDSNQDARRRSVNEDDNPPSPIGG**    990 |
| SS_PSIPRED |  | EEEE      EEEEEE  EEEEEEEEE   EEEEE                     HHHHHHHH    HHHHH |
| SS_SPIDER2 |  | EEEEEEEE   EEEEEE  EEEEEEEEE   EEEEEE                E   HHHHHHHHHHHHHHHHH |
| SS_PSSPRED |  | HHHHH       HHHHHHHHHHEEEEEEE   EEEEE                     HHHHHHHHH    HHHH |
| SS_DEEPCNF |  | EEEEE      EEEEEE    EEEEEE    EEEEE                    HHHHHHHHH    HHHHH |
| DO_DISOPRED3 |  | DDDDDDDDDDDD |
| DO_SPOTD |  | DDDDDDDDDDDDDDDD |
| DO_IUPRED |  | D    DDDDDDDDDDDDDDDDDDDDD |
|  | | |
|  | | |
|  | | |
|  | | |
|  | | |
| AA_QUERY | 991 | **DMMDSLISQLQPPPQQQPFPKQPGTSGAYPLTSPPTSYHSTVNQSPSMMHTQSPGNLHAASSPSGALRAPSPASFVPTPPPSSHGISIGP**    1080 |
| SS_PSIPRED |  |  |
| SS_SPIDER2 |  | H |
| SS_PSSPRED |  | HHHHHHHHH |
| SS_DEEPCNF |  | HHHHHHH |
| DO_DISOPRED3 |  | D  DDDDDDDDDDDDDDDDDDDDDDDDDDDDDDDDDDDDDD   DDDDDDDDDDDDDDDDDDDDDDDDDDDD   D |
| DO_SPOTD |  | DDDDDDDDDDDDDDDDDDDDDDDDDDDDDDDDDDDDDDDDDDDDDDDDDDDDDDDDDDDDDDDDDDDDDDDDDDDDDDDDDDDDDDDDDD |
| DO_IUPRED |  | DDDDDDDDDDDDDDDDDDDDDDDDDDDDDDDDDDDDDDDDDDDDDDDDDDDDDDDDDDDDDDDDDDDDDDDDDDDDDDDDDDDDDDDDDD |
|  | | |
|  | | |
|  | | |
|  | | |
|  | | |
| AA_QUERY | 1081 | **GASFASPHGTLDPSSPYTMVSPSGRAGNWPGSPQVSGPSPAARMPGMSPANPSLHSPVPDASHSPRAGTSSQTMPTNMPPPRKLPQRSWA**    1170 |
| SS_PSIPRED |  |  |
| SS_SPIDER2 |  |  |
| SS_PSSPRED |  | EE                                                                    HHH |
| SS_DEEPCNF |  |  |
| DO_DISOPRED3 |  | DDDDDDDDDDDDDDDDDDDDDDDDDDDDDDDDDDDDDDDDDDDDDDDDDDDDDDDD |
| DO_SPOTD |  | DDDDDDDDDDDDDDDDDDDDDDDDDDDDDDDDDDDDDDDDDDDDDDDDDDDDDDDDDDDDDDDDDDDDDDDDDDDDDDDDDDD |
| DO_IUPRED |  | DDDDDDDDDDDDDDDDDDDDDDDDDDDDDDDDDDDDDDDDDDDDDDDDDDDDDDDDDDDDDDDDDDDDDDDDDDDDDDDDDDDDDDDDDD |
|  | | |
|  | | |
|  | | |
|  | | |
|  | | |
| AA_QUERY | 1171 | **ASIPTILTHSALNILLLPSPTPGLVPGLAGSYLCSPLERFLGSVIMRRHLQRIIQQETLQLINSNEPGVIMFKTDALKCRVALSPKTNQT**    1260 |
| SS_PSIPRED |  | HHHHHHHH                   HHHHHHHHHHHHHHHHHHHHH   EE       EEEEE  EEEEEEEE      E |
| SS_SPIDER2 |  | HHHHHH                     HHHHHHHHHHHHHHHHHHHH HHHHH       EEEEE   EEEEEEE     EE |
| SS_PSSPRED |  | HHHHHHHHHHHHHHEEE            H HHHHHHHHHHHHHHHHHHHHHHHHHHHHHHHH     EEEEEE   EEEEE       E |
| SS_DEEPCNF |  | HHHHHHHHHH                    HHHHHHHHHHHHHHHHHHHHHHHH        EEEEEEE   EEEEEE      E |
| DO_DISOPRED3 |  |  |
| DO_SPOTD |  |  |
| DO_IUPRED |  |  |
|  | | |
|  | | |
|  | | |
|  | | |
|  | | |
| AA_QUERY | 1261 | **LQLKVTPENAGQWKPDELQVLEKFFETRVAGPPFKANTLIAFTKLLGAPTHILRDCVHIMKLELFPDQATQLKWNVQFCLTIPPSAPPIA**    1350 |
| SS_PSIPRED |  | EEEEE         HHHHHHHHHHHHHH       HHHHHHHHHHH   HHHHHHHHHHH HH         EEEEEEEEE |
| SS_SPIDER2 |  | EEEEE         HHHHHHHHHHHHHHH      HHHHHHHHHHH   HHHHHHHHHHHHHH         EEEEEEEEEE |
| SS_PSSPRED |  | EEEEE          HHHHHHHHHHHHH       HHHHHHHHHHH  HHHHHHHHHHHHHHHH  HH HHHHEEEEEEEE |
| SS_DEEPCNF |  | EEEEE          HHHHHHHHHHH         HHHHHHHHHHH    EEE   EEEEEEEE        EEEEEEEEEE |
| DO_DISOPRED3 |  |  |
| DO_SPOTD |  |  |
| DO_IUPRED |  | DDD |
|  | | |
|  | | |
|  | | |
|  | | |
|  | | |
| AA_QUERY | 1351 | **PPGTPAVVLKSKMLFFLQLTQKTSVPPQEPVSIIVPIIYDMASGTTQQADIPRQQNSSVAAPMMVSNILKRFAEMNPPRQGECTIFAAVR**    1440 |
| SS_PSIPRED |  | EEE    EEEEEEEE           EEEEEEEEE     EEE           HHHHHHHHHHHHHHHH         HHHHHH |
| SS_SPIDER2 |  | EEEE   EEEEEEEEE          EEEEEEEEE    EEEE           HHHHHHHHHHHHHHHH         HHHHHH |
| SS_PSSPRED |  | EEEEHHHHHHHHHHH           EEEHHHHHH                  HHHHHHHHHHHHHHHHH       EHHHHHHH |
| SS_DEEPCNF |  | EEEE   EEEEEEEE           EEEEEEEEE                    HHHHHHHHHHHHHH          HHHHHH |
| DO_DISOPRED3 |  | DD D                  DDDDDDDDDDDDDDDD |
| DO_SPOTD |  |  |
| DO_IUPRED |  | D                                               DDDDDDDD |
|  | | |
|  | | |
|  | | |
|  | | |
|  | | |
| AA_QUERY | 1441 | **DLMANLTLPPGGRP**    1454 |
| SS_PSIPRED |  | HHHHH |
| SS_SPIDER2 |  | HHHHH |
| SS_PSSPRED |  | HHHHH |
| SS_DEEPCNF |  | HHHH |
| DO_DISOPRED3 |  | DDDDDD |
| DO_SPOTD |  | DDDD |
| DO_IUPRED |  | DDDDDDDD |
|  | | |
|  | | |
|  | | |
|  | | |
|  | | |

SS = Secondary Structure; H = Alpha-helix; E= Beta-strand; CC = Coiled coils; D, DO = Disordered Region
