# Supplementary material for: N-terminal half of MED14 is critical for Mediator-RNA polymerase II interaction and the resulting transcription
Source: J Biol Chem. 2025 Oct 17;301(12):110837. doi: 10.1016/j.jbc.2025.110837 (PMC12719649; doi:10.1016/j.jbc.2025.110837)
Supplement: Supplementary Figure and Table Legends [file mmc3.docx]

## Supplementary Figure Legends

**Supplementary Figure S1. Gel filtration traces for recombinant core Mediator subcomplexes**

Recombinant complexes are purified as depicted in Figure 1b-c and run over 10 ml of Superose 6 gel filtration column. Chromatography traces of three core Mediator subcomplexes are represented.

## Supplementary Figure S2. Purification of Gdown1-free Pol II and MED14 interaction with Pol II

**(a)** Purification of Gdown1-free Pol II. Nuclear extract from HeLa cells stably expressing f:RPB9 was run on HiTrap Q HP (anion-exchange, step 1) followed by HiTrap Heparin (cation-exchange, step 2) columns. Pol II fractions mostly cleared from Gdown1 were pooled together and further purified over M2 agarose affinity resin (step 3). The right panel shows SDS-PAGE followed by silver staining of purified Pol II. **(b)** Western blot analysis of recombinant MED14 and Pol II interaction assay. Equimolar amounts of Pol II (50 nM) were conjugated to α-8WG16-coupled (against RPB1) beads. 50 nM each of recombinant f:MED14-NTD, f:MED14-CTD, and f:MED14 was added to each reaction, and the co-purified proteins were assayed by western blot. Lanes 1-3 show the input of recombinant MED14 fragments. Lane 4 shows the input of Pol II. Lanes 5-7 show the precipitated proteins indicating interactions with the bead-conjugated Pol II.

## Supplementary Figure S3. Expression of FLAG-, His-, and HA-tagged RPB subunits in insect cells

**(a)** SDS-PAGE (Coomassie staining) analysis of purified f:RPB1. **(b)** Superose 6 gel-filtration spectra and the corresponding western blot of His:RPB1 eluates. **(c)** Monomer and dimer eluates of His:RPB1 from (b) were pooled together and further purified using Nickel resin. **(d)** Western blot of individually cloned and purified His:RPB3, His:RPB5, His:RPB6, His:RPB7, His:RPB9, His:RPB12, HA:RPB4, HA:RPB8, HA:RPB10, and HA:RPB11.

## Supplementary Figure S4. Characterization of recombinant Pol II subunit interactions with reconstituted human core Mediator subcomplexes

**(a-f)** Western blot analysis of recombinant Mediator subcomplexes and RNA Pol II subunit interaction assays. Equimolar amounts (50 nM each) of either recombinant human Mediator, MED14- NTD+H+M+MED26, MED14+H+M+MED26, or MED14-NTD+H were conjugated to either α-MED30- **(a)** or α-FLAG-coupled **(b-f)** beads. Recombinant RPB subunits from Supplementary Figure S3 were added to each reaction, and the co-purified proteins were assayed by western blot. Red asterisks show MED17 antibody from prior blotting that could not be completely stripped off.

## Supplementary Figure S5. Pol II-MED14 interaction as illustrated on PDB Database

PDB ID: 5U0S revealed the MED14-NTD region (RM1) to be in close proximity with the CTD linker region of RPB1. Most of the disordered CTD was not characterized in the structure.

**Supplementary Table Legends**

**Supplementary Table S1. LC-MS/MS of RPB1 and RPB1-CTD peptides and phosphorylation status (related to Figure 3).**

**Sheet 1** lists the top 34 proteins identified by mass spectrometry, with reconstituted Mediator subunits highlighted in green and recombinant RPB1 (full-length or CTD-only) highlighted in purple; lanes J-M show reconstituted Mediator pulling down either RPB1 or CTD-RPB1. **Sheet 2** provides the peptide table (sequence, modification, and MS1 areas) for lanes Z-AC, along with a summary bar chart showing global CTD phosphorylation (Σ phospho-CTD areas ÷ Σ all CTD peptides) and sequence-specific occupancy for the peptide YSPTSPTYSPTSPK. In the chart, unmodified CTD peptides are shown in gray, phosphorylated CTD peptides in blue, and values not detected are labeled N.D. The key results are that MED14+H+M and MED14-NTD+H+M with full-length RPB1 had global CTD phosphorylation of 0%, while lane MED14-NTD with isolated RPB1-CTD showed a low level of phosphorylation (≈0.28%) and a sequence-specific occupancy of 0.78% for YSPTSPTYSPTSPK. Full acquisition and calculation details are described in Materials and Methods (Mass spectrometry analysis).

**Supplementary Table S2. HHpred secondary structure prediction of *S. pombe* and human MED14**

Sequence and secondary structure similarities between *S. pombe* and human MED14 were assessed using HHpred.
