# Supplementary material for: N-terminal half of MED14 is critical for Mediator-RNA polymerase II interaction and the resulting transcription
Source: J Biol Chem. 2025 Oct 17;301(12):110837. doi: 10.1016/j.jbc.2025.110837 (PMC12719649; doi:10.1016/j.jbc.2025.110837)

Gel Filtration Traces of f:MED14+H+M+MED26, f:MED14-NTD+H+M+MED26 and f:MED14-CTD+H+M+MED26

Supplementary Figure S1

Superose 6 column (10ml)

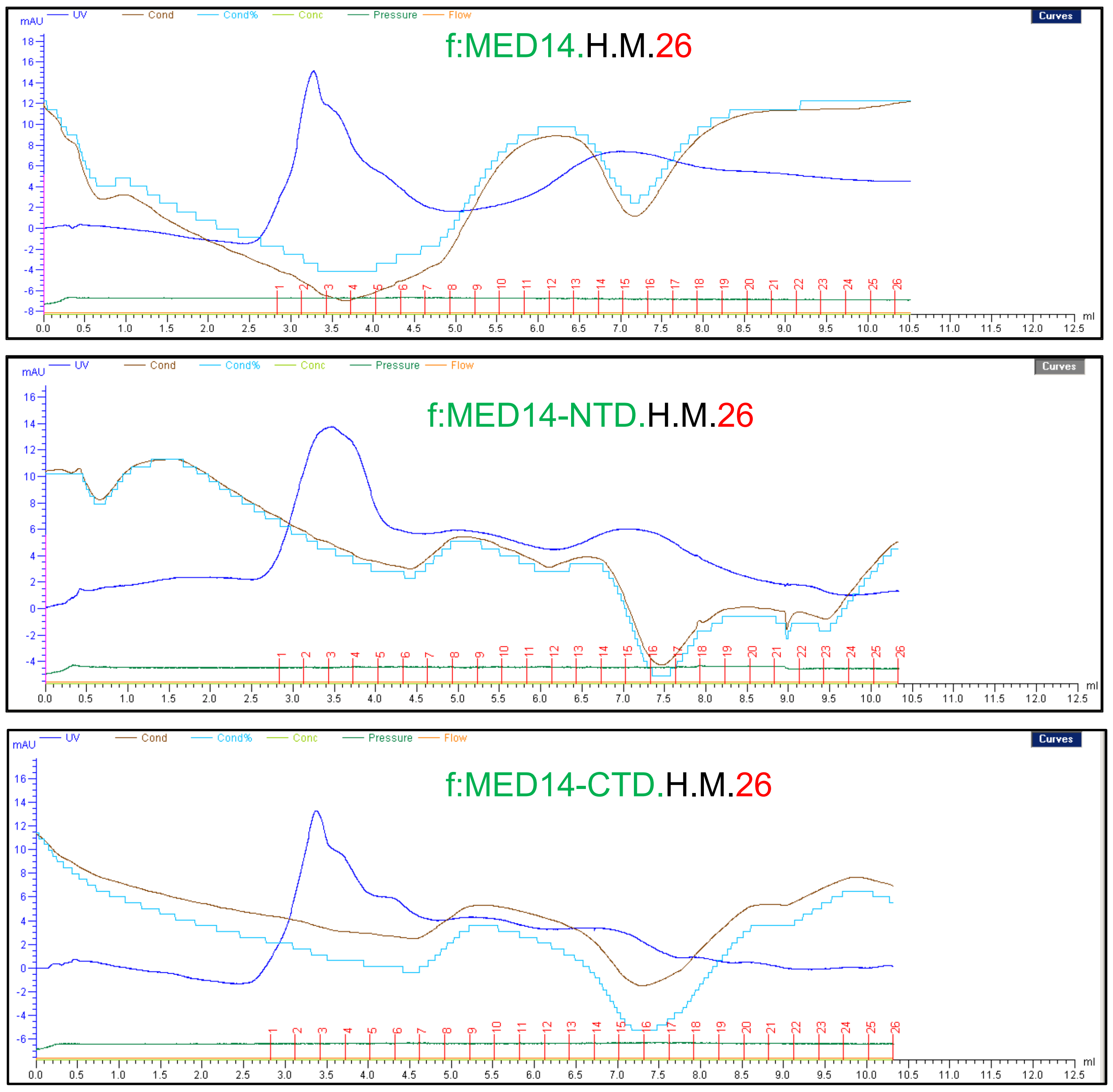

Purification of Pol II and IP with Recombinant MED14 Variants

Supplementary Figure S2

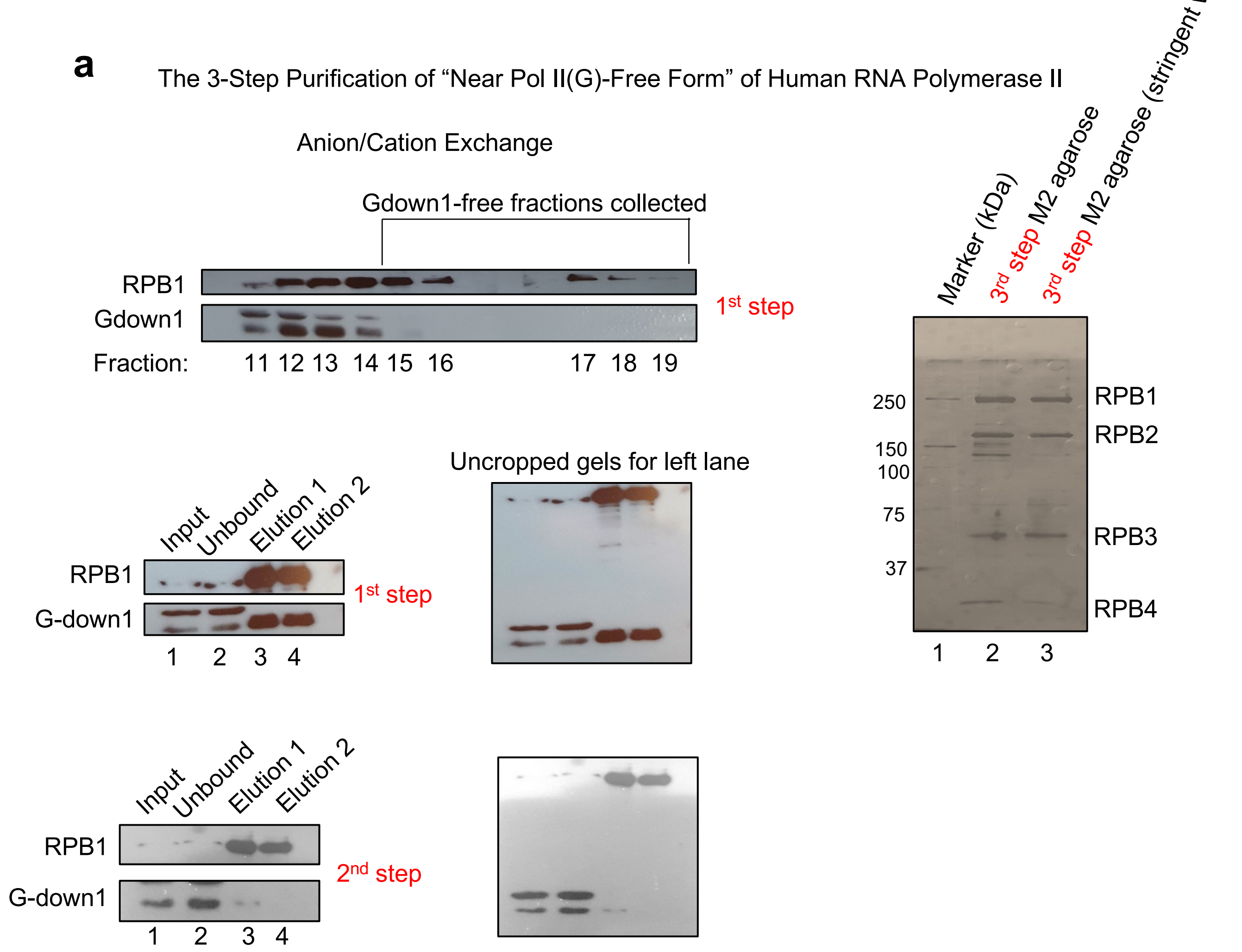

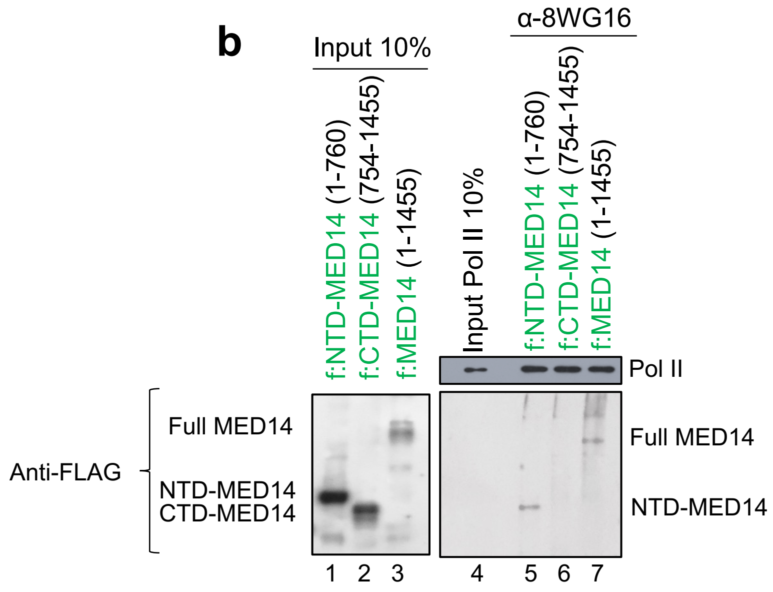

Recombinant FLAG-, His-, and HA-Tagged Pol II Subunits

Supplementary Figure S3

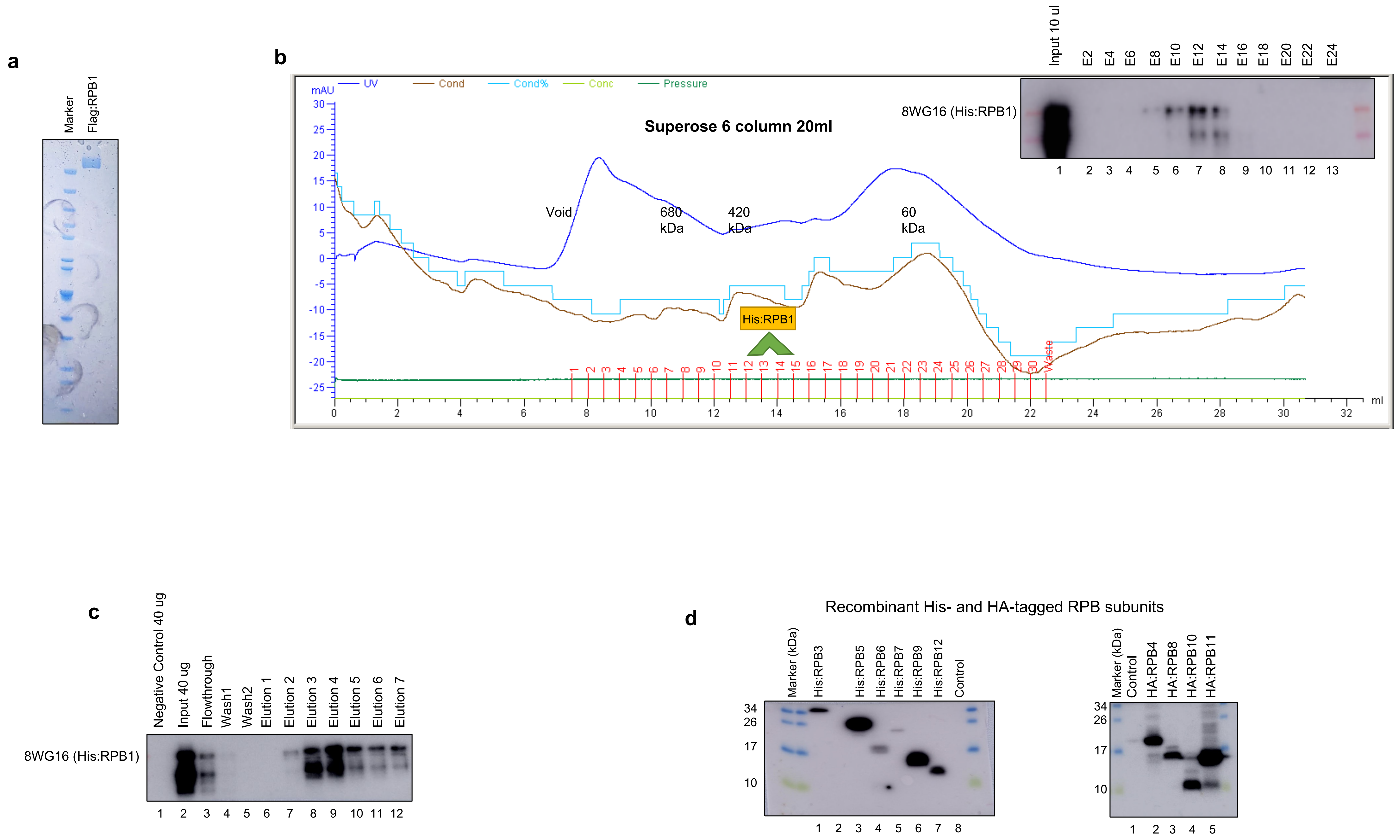

Characterization of MED14-NTD Human Core Interaction with Recombinant Pol II Subunits

Supplementary Figure S4

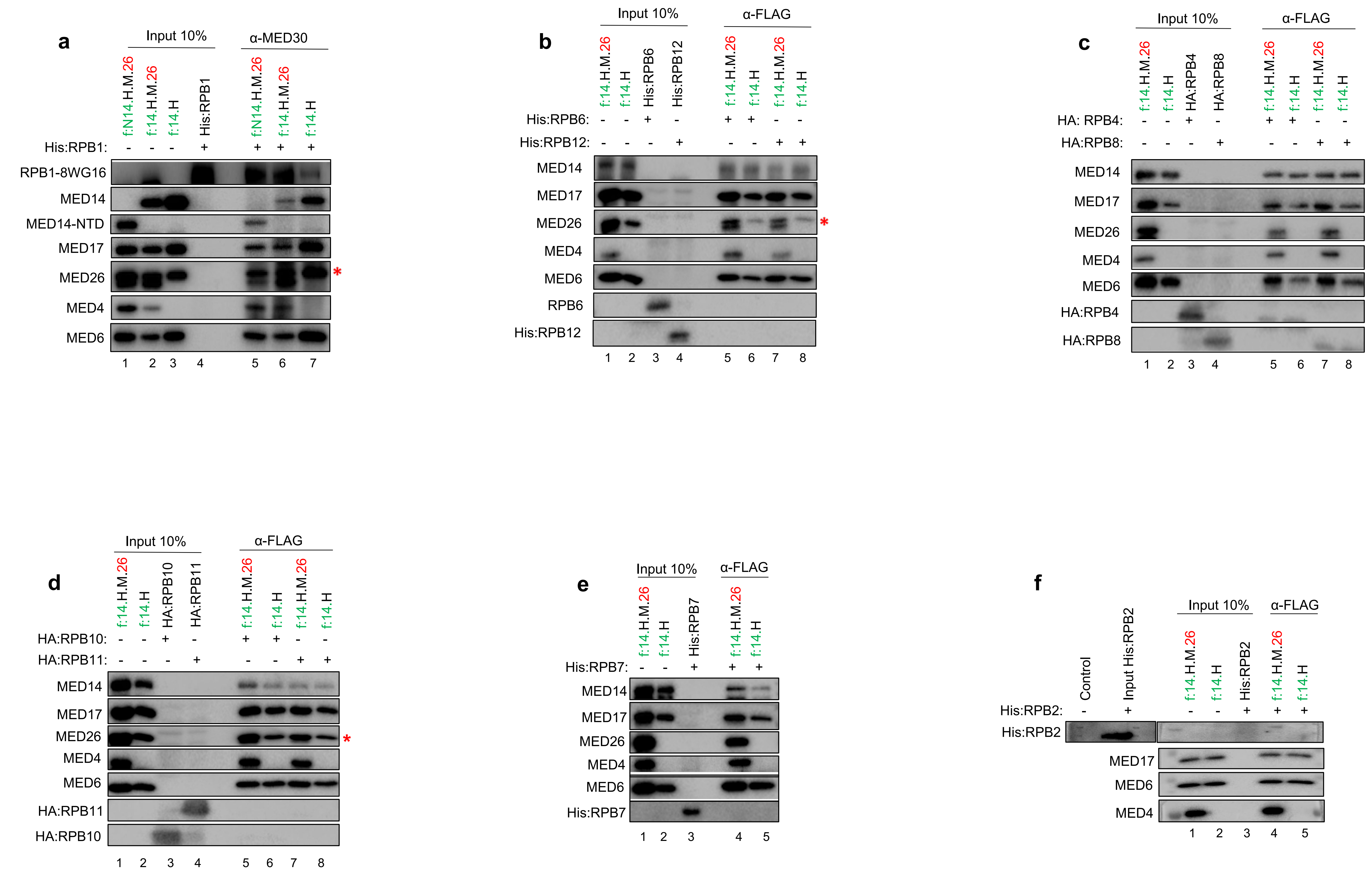

NTD-MED14 is Critical for Pol II Interaction

Supplementary Figure S5

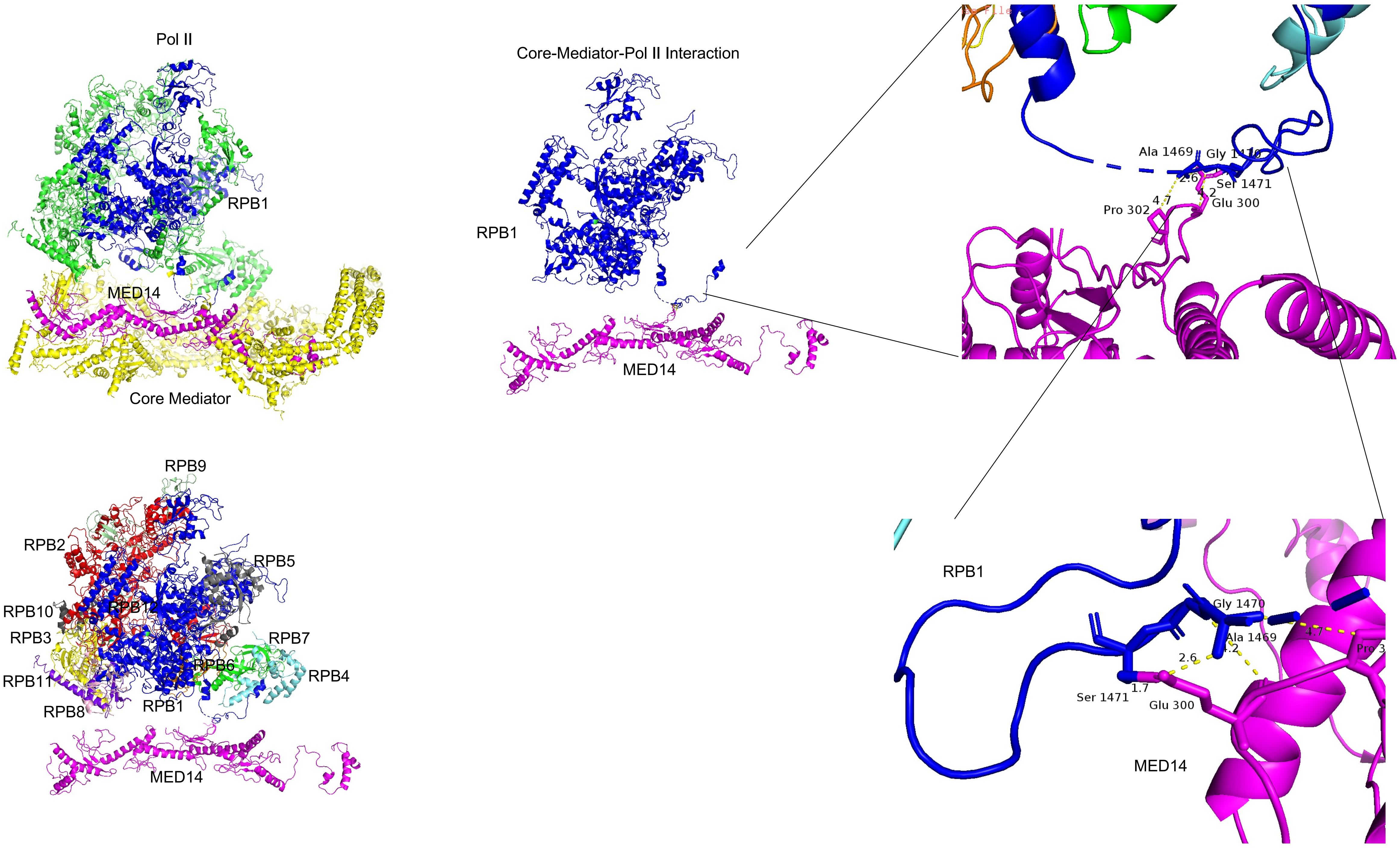

Supplement: Supplementary Table S2 [file mmc5.pdf]
